# Health Care Professionals’ Perceptions of AI in Clinical Practice: Productivity, Enjoyment, and Pay

**DOI:** 10.2196/90100

**Published:** 2026-09-02

**Authors:** Michael L Chen, Jiyeong Kim, Mariana Ramirez Posada, David Entwistle, Isabella de Vere Hunt, Yifan Li, Xue Li, Nathanael J Fast, Fatima Rodriquez, Albert Chiou, Euan Ashley, Eleni Linos

**Affiliations:** 1 Stanford University, 269 Campus Drive, Stanford, CA, 94305, United States, 1 6507250452; 2 University of Oxford, Oxford, England, United Kingdom; 3 University of Hong Kong, Hong Kong, China (Hong Kong); 4 University of Southern California, Los Angeles, CA, United States

**Keywords:** artificial intelligence, perception of health care professions, AI adoption in medicine, AI and job satisfaction, AI and productivity

## Abstract

Despite widespread discussion of opportunities and risks about AI in medicine, few health care professionals routinely used AI during this study period. Physicians and men were more likely to report frequent AI use, and frequent AI users more often reported positive sentiments about AI’s future impacts on pay, enjoyment, and productivity at work. The youngest professionals (≤29 years) were less engaged and more skeptical about AI’s future benefits. These findings highlight a gap between AI’s promise and medical practice, as adoption varies by role, experience, and demographics.

## Introduction

Artificial intelligence (AI) is rapidly being integrated into medicine [1]. Early studies have demonstrated the potential of AI to enhance diagnostic accuracy, reduce clinician workload, and improve patient outcomes [2]. At the same time, concerns persist regarding algorithmic bias, lack of transparency, regulatory barriers, and the ethical implications of replacing or supplementing human judgment [3,4]. Despite widespread discussion of these opportunities and risks, how health care professionals perceive AI’s future role in clinical practice remains understudied. Previous surveys suggest mixed attitudes, with enthusiasm for improved disease screening, greater efficiency, and reducing repetitive tasks, and skepticism about liability for errors, and erosion of the clinician’s role [5,6]. However, studies were limited to single institutions, specific specialties, or narrow clinical contexts, leaving uncertainty about differences across job categories, genders, or age groups [7].

We sought to address this gap by surveying health care professionals across 2 large US academic health systems to characterize AI use in routine work, evaluate trust in AI, and explore how perceptions of AI’s future impact vary by professional and demographic groups. We aim to inform strategies for equitable and effective implementation of AI in clinical settings.

## Methods

We conducted a cross-sectional survey of health care professionals within 2 large academic health care systems (Stanford Health Care and Wake Forest University/Advocate Health) (September 23, 2024, to February 28, 2025). We recruited health care professionals through multiple overlapping email lists to maximize reach; however, this approach precluded calculation of a precise response rate. Survey questions (Multimedia Appendix 1), based on the USC Neely-UAS AI Index (University of Southern California, Neely Understanding America Study Artificial Intelligence Index) [8], were optional, and we treated the missing data using the missing-at-random assumption. Topics included frequency of AI use, perceptions toward the use of AI in health care, and perceived impact of AI on enjoyment (AI will increase/decrease/not change my enjoyment), accomplishment (AI will increase/decrease/not change my productivity), and pay (AI will increase/decrease/not change my pay). We performed descriptive analyses using *χ*² tests. To identify factors associated with perceptions of AI by frequency of use (use AI frequently, rarely, or sometimes [=reference group]), we developed multinomial logistic regression models to obtain odds ratios (ORs) and 95% CIs, adjusting for job (physician vs nonphysician), gender (women vs men), and age (≤29, 30-34, 35-39, 40-44, 45-49, 50-54, 55-59, 60-64, and ≥65 years). Statistical significance was determined at *P*<.05 (Python 3.10 in Google Colab, Google LLC, Mountain View, California). The Stanford University Institutional Review Board deemed this study exempt.

## Results

**Figure 1.**
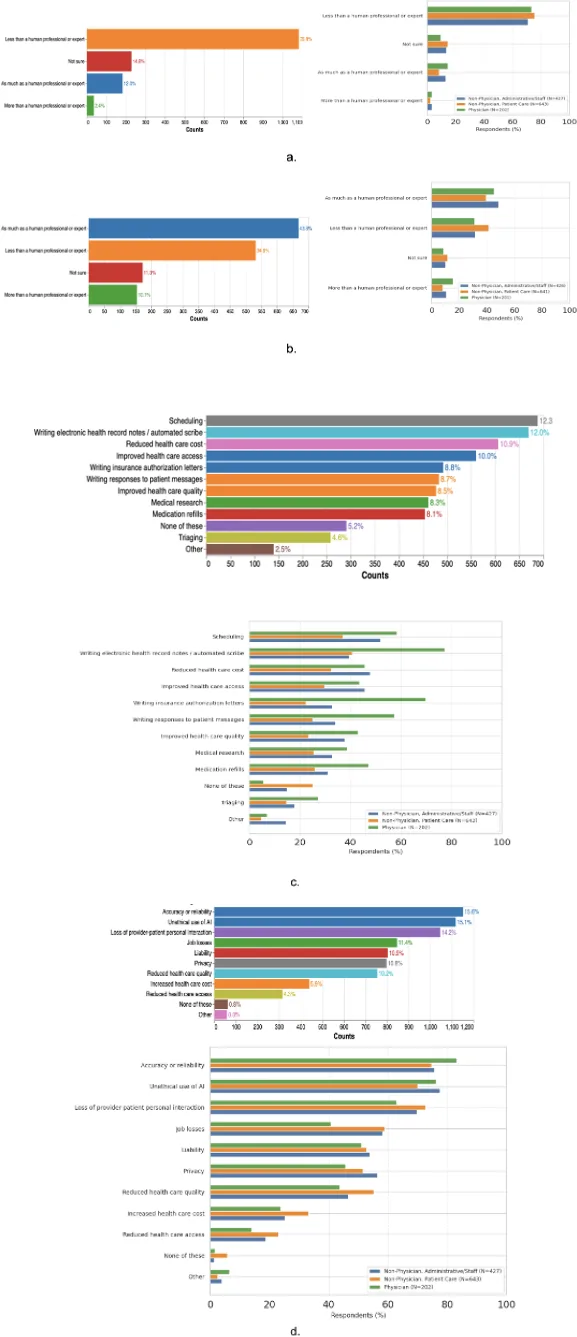
Differences in perceptions of health care professionals toward the use of AI in health care by job title. (A) How much would you trust AI in giving a health diagnosis? (B) How much would you trust AI in designing a diet and exercise program? (C) In which of the following areas does using AI in health care excite you? (select all that apply). (D) In which of the following areas does using AI in health care concern you? (select all that apply).

A total of 2235 participants responded, and of respondents who reported job status, gender, and age, 79.2% (1158/1463) were younger than 55 years, 74.4% (1086/1460) were women, and 13.9% (203/1465) were physicians, while nonphysicians included direct patient care (644/1262, 51.0%) and administrative roles (431/1262, 34.2%). Overall, 10.6% (162/1525) of health care professionals were frequent users of AI in their work (using AI every day), and 65.0% (991/1525) were rare users. The frequency of AI use and the perceived impact of AI on pay, enjoyment, and productivity at work differed by job, gender, and age. Physicians (50/201, 24.9% vs nonphysicians 102/1254, 8.1%; *P*<.001) and men (60/343, 17.5% vs women 92/1080, 8.5%; *P*<.001) were more likely to be everyday users. The youngest (≤29 years) rarely used AI at work (129/172, 75.0%) and perceived AI negatively in productivity, enjoyment, and pay at work (*P*<.001). More than 70% of respondents showed higher trust in human experts in giving a health diagnosis (1083/1530, 70.8%) than in AI. However, 53.9% (822/1526) had trust in AI in designing diet and exercise programs as much as or more than human experts (Figure 1).

We specified AI in this survey as large language models, or a type of AI that generates human-like text (eg, ChatGPT, Google Bard, GPT-4V, Med-PaLM).

Physicians showed more trust in AI in giving a health diagnosis (physicians 35/202, 17.3% vs nonphysicians 171/1256, 13.6%) and designing diet and exercise programs (physicians 122/201, 60.7% vs nonphysicians 667/1254, 53.2%). The concerns included accuracy or reliability, unethical use of AI, and loss of provider-patient personal interaction. Physicians were excited to use AI for writing electronic health records notes/automated scribe, while nonphysicians were mostly excited about scheduling. Higher frequency of AI use was significantly associated with improved perception of AI on future pay (OR 2.44, 95% CI 1.42‐4.20 vs no change; *P*<.001), enjoyment at work (OR 3.73, 2.28‐6.12 vs no change; *P*<.001), and productivity (OR 1.95, 1.12‐3.42 vs no change; *P*=.02) (Table 1).

**Table 1.** The association of the frequency of the use and the perceived impact of AI at work.

	Frequent users^a^		Rare users^a^	
	aOR^b^ (95% CI)	*P* value	aOR^b^ (95% CI)	*P* value
Perceived impact on pay^c^ (n=1409)				
AI will increase my pay	2.44 (1.42‐4.20)	<.001	0.53 (0.33‐0.85)	.01
AI will decrease my pay	0.90 (0.55‐1.49)	.69	2.19 (1.64‐2.92)	<.001
AI will not change my pay	Reference		Reference	
Perceived impact on enjoyment^d^ (n=1405)				
AI will increase my enjoyment at work	3.73 (2.28‐6.12)	<.001	0.29 (0.22‐0.39)	<.001
AI will decrease my enjoyment at work	2.08 (0.74‐5.82)	.16	2.93 (1.72‐4.99)	<.001
AI will not change my enjoyment at work	Reference		Reference	
Perceived impact on productivity^e^ (n=1403)				
AI will increase my productivity at work	1.95 (1.12‐3.42)	.02	0.22 (0.16‐0.30)	<.001
AI will decrease my productivity at work	1.05 (0.21‐5.41)	.95	1.74 (0.81‐3.74)	.16
AI will not change my productivity at work	Reference		Reference	

a Frequent users (n=162): use AI every day; rare users (n=991): use AI rarely or less than once a month; sometimes users (n=372; reference group): use AI once a week or once a month.

b aOR: adjusted odds ratio (adjusted for job, gender, and age).

c Question: How do you think AI technologies might influence how much you get paid over the next 5 years?”

d Question: Thinking of your current job, how do you think AI technologies might change how much you enjoy your job in the next 5 years?

e Question: How do you think AI technologies might change how much you can accomplish in a typical workday over the next 5 years? (Full frequency is available in the Multimedia Appendix 2.)

## Discussion

Overall, a small proportion of health care professionals during this study period incorporated AI regularly in routine work. We hypothesize this reflects the early stages of formal AI programs, nascent use cases for direct patient care, and barriers relating to AI regulatory requirements [1,9]. Physicians and men were more likely to report frequent AI use, while the youngest professionals (≤29 years) were less engaged and more skeptical about AI’s future benefits for pay, enjoyment, and productivity, suggesting that AI adoption and perception are shaped by role (eg, different clinical responsibilities, limited exposure to AI at work given their early career), and demographic factors. We suggest that further research explores the effect of differential access, job responsibilities, or levels of training on workplace AI use.

Frequent AI users were more likely to report a positive sentiment on pay, enjoyment, and productivity, possibly reflecting a self-reinforcing cycle of exposure in building familiarity and trust. Further research should examine whether attitudes drive usage or interactions change sentiments to inform adoption strategies, such as AI onboarding and clarity on institutional policies.

Consistent with previous studies, clinicians expressed strong concerns about accuracy and reliability and erosion of the provider-patient relationship [3,10]. Addressing these concerns will require technical improvements and organizational strategies that preserve human-centered care. Enthusiasm for automated documentation among physicians and scheduling support among nonphysicians highlights the need for tailored solutions, and willingness to use AI for designing diet and exercise programs suggests adoption may gain traction in lower-risk domains.

Study limitations include surveying within only 2 academic health systems, which may not represent other health care environments, and the optional nature of the survey, which may have introduced selection bias. Additionally, the nonphysician category was heterogeneous, limiting our ability to identify subgroup-specific trends.

In conclusion, although most health care professionals rarely use AI, greater exposure increases optimism. Ensuring equitable access, addressing subgroup concerns, and building trust through training with institutional support is critical.

## Supplementary material

10.2196/90100 Multimedia Appendix 1 Additional information.

10.2196/90100 Multimedia Appendix 2 Survey questionnaires.

## Abbreviations

OR: odds ratio
USC Neely-UAS AI Index: University of Southern California, Neely Understanding America Study Artificial Intelligence Index
