# Supplementary material for: Health Care Professionals’ Perceptions of AI in Clinical Practice: Productivity, Enjoyment, and Pay
Source: JMIR Form Res. 2026 Sep 2;10:e90100. doi: 10.2196/90100 (PMC13537275; doi:10.2196/90100)
Supplement: Supplementary file 1 [file formative-v10-e90100-s002.docx]

AI Perspectives

Start of Block: Questionnaire

T3
**Artificial intelligence (AI)** in this survey refers to technology capable of analyzing text, sound, and/or images – tasks traditionally performed by humans.
 
This survey also asks questions specifically about large language models, or a **type of AI that generates human-like text** (eg, ChatGPT, Google Bard, GPT-4V, Med-PaLM).

T4
**Section 1. AI Use**

N1 What do you use AI applications such as ChatGPT, Google Bard/Gemini, or Bing Chat for in your work? Please select all that apply.

- Out of curiosity (6)
- To gather information or explore details about a specific health condition or treatment (1)
- To create content for social media (7)
- To improve communications (for instance, help in writing emails, letters, etc.) (8)
- To assist in organizational tasks (3)
- To learn something new about the world (4)
- None of these (5)

Q2 For which of the following tasks do you utilize AI in your work? Select all that apply.

- Diagnosis (13)
- Treatment/Management Plan (1)
- Writing electronic health record notes (8)
- Writing responses to patient messages (14)
- Writing insurance authorization letters (10)
- Creating patient handouts (15)
- Other (9)
- None of these (11)

N3 How **often** do you use AI tools in your work?

- Rarely (1)
- Less than once a month (6)
- Once or a few times per month (2)
- Once or a few times per week (3)
- Every day (4)

T5 **Section 2. AI Perspectives**

N4 How **useful** do you find AI applications such as ChatGPT, Google Bard/Gemini, or Bing Chat?

- Not at all useful (1)
- Not very useful (2)
- Somewhat useful (3)
- Very useful (4)
- Extremely useful (6)
- No opinion (5)

N5 How **harmful** do you find AI applications such as ChatGPT, Google Bard/Gemini, or Bing Chat?

- Not at all harmful (1)
- Not very harmful (2)
- Somewhat harmful (3)
- Very harmful (4)
- Extremely harmful (6)
- No opinion (5)

Q6 How **aligned** do you find your hospital's AI strategy is with your needs and priorities?

- Not at all aligned (1)
- Not very aligned (2)
- Somewhat aligned (3)
- Very aligned (4)
- Extremely aligned (5)
- No opinion (6)

N7 How much would you **trust** AI in the following daily life activities?

|  | Less than a human professional or expert (1) | As much as a human professional or expert (2) | More than a human professional or expert (3) | Not sure (4) |
| --- | --- | --- | --- | --- |
| Giving a health diagnosis (1) |  |  |  |  |
| Providing financial advice (4) |  |  |  |  |
| Designing a diet and exercise program (2) |  |  |  |  |
| Making travel reservations (5) |  |  |  |  |
| Driving a taxi cab (6) |  |  |  |  |

N8 Thinking of your current job, how do you think AI technologies might change how much you can **accomplish in a typical workday** over the next 5 years?

- AI will increase how much I can accomplish in a workday (1)
- AI will decrease how much I can accomplish in a workday (2)
- AI will not change how much I can accomplish in a workday (3)

N9 Thinking of your current job, how do you think AI technologies might change how much you **enjoy your job** in the next 5 years?

- AI will increase the enjoyment I get from my job (1)
- AI will decrease the enjoyment I get from my job (2)
- AI will not change the enjoyment I get from my job (3)

N10 Thinking of your current job, how do you think AI technologies might influence how much you **get paid** over the next 5 years?

- AI will increase my pay (1)
- AI will decrease my pay (2)
- AI will not change my pay (3)

N11 Now, think of the tasks that you typically perform as part of your job. On a scale from 0 to 100, where 0 means none and 100 means all of them, what **portion** of these tasks do you think could be performed either **partially or entirely by AI** over the next 5 years?

|  | 0 | 10 | 20 | 30 | 40 | 50 | 60 | 70 | 80 | 90 | 100 |
| --- | --- | --- | --- | --- | --- | --- | --- | --- | --- | --- | --- |

| (1) |  |
| --- | --- |

N12 On a scale from 0 to 100, where 0 means absolutely no chance and 100 means absolutely certain, what are the **chances** that you will lose your job because it is **replaced by AI** in the next 5 years?

|  | 0 | 10 | 20 | 30 | 40 | 50 | 60 | 70 | 80 | 90 | 100 |
| --- | --- | --- | --- | --- | --- | --- | --- | --- | --- | --- | --- |

| (1) |  |
| --- | --- |

Q13 In which of the following areas does using AI in healthcare **excite you**? Select all that apply.

- Writing electronic health record notes / automated scribe (10)
- Writing responses to patient messages (11)
- Writing insurance authorization letters (3)
- Triaging (9)
- Medication refills (14)
- Scheduling (13)
- Medical research (12)
- Improved health care quality (4)
- Improved health care access (2)
- Reduced health care cost (5)
- Other (8)
- None of these (6)

Q14 In which of the following areas does using AI in healthcare **concern you**? Select all that apply.

- Loss of provider-patient personal interaction (4)
- Unethical use of AI (6)
- Job losses (7)
- Privacy (8)
- Liability (5)
- Accuracy or reliability (9)
- Reduced health care quality (1)
- Reduced health care access (11)
- Increased health care cost (12)
- Other (2)
- None of these (10)

Q15 Are there **promising areas** for AI in healthcare that were not asked about? If so, please describe.

________________________________________________________________

________________________________________________________________

________________________________________________________________

________________________________________________________________

________________________________________________________________

Q16 Do you have **concerns** about the increasing use of AI in healthcare that were not asked about? If so, please describe.

________________________________________________________________

________________________________________________________________

________________________________________________________________

________________________________________________________________

________________________________________________________________

Q17 Are you a physician (i.e., attending, fellow, or resident)?

- Yes (1)
- No (2)

End of Block: Questionnaire

Start of Block: General

Q18 In which country do you currently practice?

▼ United States of America (187) ... Zimbabwe (1357)

Q19 In which state do you currently practice?

▼ Alabama (1) ... I do not reside in the United States (53)

Display this question:

If Are you a physician (i.e., attending, fellow, or resident)? != Yes

Q20 Which of the following best describes your role?

- Primarily direct patient care (10)
- Primarily administrative or staff role (13)
- Other (please specifiy) (4) __________________________________________________

Display this question:

If Are you a physician (i.e., attending, fellow, or resident)? = Yes

Q21 Which of the following best describes your medical specialty?

- Anesthesia (1)
- Dermatology (4)
- Emergency Medicine (5)
- Family Medicine (2)
- Internal Medicine (3)
- Neurology (6)
- Obstetrics-gynecology (7)
- Ophthalmology (8)
- Pathology (9)
- Pediatrics (10)
- Psychiatry (11)
- Radiology (12)
- Surgery (13)
- Other (please specify) (14) __________________________________________________

Q22 Which of the following best describes your work setting?

- Private Practice (1)
- Academic (4)
- Public Hospital (3)
- Health Maintenance Organization (HMO) (2)
- Veterans affairs/Military (7)
- Not currently working (8)
- Other (please specify): (5) __________________________________________________

Q23 What type of community do you predominantly serve?

- Rural (1)
- Suburban (2)
- Urban (3)

Q24 Do you work with vulnerable populations?

- Yes (1)
- No (2)

Q25 What is your gender identity?

- Woman (1)
- Man (9)
- Self-describe: (4) __________________________________________________

Q26 What is your age?

- 29 years old or younger (1)
- 30-34 years old (9)
- 35-39 years old (2)
- 40-44 years old (3)
- 45-49 years old (4)
- 50-54 years old (5)
- 55-59 years old (6)
- 60-64 years old (7)
- 65+ years old (8)

Q27 Which of the following best describes your race? Select all that apply.

- American Indian or Alaska Native (1)
- Asian (2)
- Black or African American (3)
- Native Hawaiian or Pacific Islander (4)
- White (5)
- Other (6) __________________________________________________

Q28 Do you identify as Hispanic or Latino?

- Yes (1)
- No (2)

End of Block: General
